# Editorial: Microbial prospecting and biomaterials

**DOI:** 10.3389/fmicb.2024.1540191

**Published:** 2025-01-07

**Authors:** Selvakumar Rajendran, Karthik Loganathan, Ketul C. Popat

**Affiliations:** ^1^Department of Nanobiotechnology, PSG Institute of Advanced Studies, Coimbatore, India; ^2^Salem Microbes Pvt Ltd, Salem, India; ^3^Department of Bioengineering, College of Engineering and Computing, George Mason University, Fairfax, VA, United States

**Keywords:** microbial prospecting, biomaterials, bioengineering, bionanomaterials, enzymes

Diversity in microbial species, ecology, metabolisms, and survival techniques provides scientists with clues to bioprospect them for various applications. Microbial bio-prospecting especially for bio-derived materials, molecules, enzymes, etc., have been studied as an alternative to various synthetic methodologies that have both economic and environmental impacts. Applying bioengineering concepts, microorganisms have been investigated as living factories for production of various biomolecules, biopolymers, enzymes, etc., on continuous basis that have potential applications in many fields (Huang et al., 2022). For example, microbial polyhydroxyalkanoates, bacterial cellulose, biogenic nanomaterials, bacterial collagens, enzymes, protein materials, etc., have superior biocompatibility, biodegradability and ease of production (intracellular/extracellular) when compared to related synthetic molecules and materials (Ghosh et al., 2021). Considering the advances in research in microbial bioprospecting and biomaterials, review and research articles were invited from researchers through this Research Topic on “*Microbial prospecting and biomaterials*.” Very interesting articles were received from various authors among which six articles were accepted for publication in “Frontiers in Microbiology” after thorough reviewing. A short note on the articles published is as follows. Thirupathi et al., reported the application of biologically synthesized Se-ZnO bimetallic nanoparticles using aqueous seaweed extract derived from *Padina boergesenii* macroalgae. The *in vitro* analysis indicated that the synthesized biometallic nanoparticle was effective against MCF-7 breast cancer and HeP G2 liver cancer cell lines with significant IC50 value. The authors suggested that the synthesized NPs by eco- and green technological method could be a potential substitute for the development of nutraceutical and therapeutic formulations against synthetic inhibitors of cancer. Similarly, Jangid et al., consolidated the bioprospecting of *Aspergillus* sp. based bioactive molecules having potential anticancer activity. The authors addressed the recent developments in drug delivery, genome mining, pharmacological profiling, role of synthetic biology and omics in discovering *Aspergillus* sp. derived anticancer compounds. Nair and Subathra Devi, reported the isolation of potential serratiopeptidase producing *Serratia marcescens* VS56 from soil samples having anti-inflammatory and clot lysing activity that could be potentially scaled up for biomedical applications. Phycocyanins derived from cyanobacteria have applications in food, cosmetics, and pharmaceutical industries and bioprospecting them remains essential. Shafiei et al., reported the methodology to achieve high pure phycocyanin from *Cyanobium* sp. isolated from waterfall in Tehran, Iran. These phycosyanin had considerable IC50 value against Calu-6 human lung cancer cell line. Veilumuthu et al., reported endophytic *Streptomyces* sp. isolated from tomato plant having genes for polyketide antibiotics. The isolate had kendomycin biosynthetic clusters for five Type I polyketide synthases. Kendomycin B was reported to be capable of microbial protein binding, membrane damage and bringing changes in nucleoid morphology in bacteria. Zhang et al., reported reverse metabolic engineering process through which Coenzyme Q10 (CoQ10; an essential ubiquinone used in pharmaceutical, food, and cosmetic additive industries) yield can be improved. They used two new mutagenesis techniques namely 12C6+ heavy-ion beam and a high-voltage prick electric field, to induce mutations in R. spheroids V-0 (V-0). They isolated and overexpressed nicotinamide adenine dinucleotide-dependent dehydrogenase (NAD) gene for enhancing productivity. The mechanism VK-2-3 CoQ10 enhancement was determined using gene knock down study. In summary, the research works reported here reinforces the capacity of using microbial prospecting approaches for developing new products and enhancing the yield of current byproducts. We strongly hope that the studies reported in this Research Topic will kindle new areas aligned to microbial prospecting for novel product development and process optimization that have not been explored yet. We thank all the authors who actively participated in this Research Topic by submitting their valuable research works. We are also grateful to all the reviewers, journal associates, and co-editors who have spent their quality time in reviewing the manuscripts for this Research Topic.
